# Validation of the Kirundi versions of brief self-rating scales for common mental disorders among children in Burundi

**DOI:** 10.1186/1471-244X-14-36

**Published:** 2014-02-12

**Authors:** Peter Ventevogel, Ivan H Komproe, Mark J Jordans, Paolo Feo, Joop TVM De Jong

**Affiliations:** 1 HealthNet TPO, Research and Development Department, Amsterdam, The Netherlands; 2 Faculty of Social Sciences, University of Utrecht, Utrecht, The Netherlands; 3 London School of Hygiene and Tropical Medicine, London, UK; 4 Child and Adolescent Neuropsychiatry Unit, Department of Neuroscience, Bambino Gesù Children’s Hospital, IRCCS, Rome, Italy; 5 University of Amsterdam, Amsterdam, The Netherlands; 6 Boston University School of Medicine, Boston, MA, USA; 7 Rhodes University, Grahamstown, South Africa

**Keywords:** Burundi, Validation, Depression, Posttraumatic stress disorder, Screening, Children

## Abstract

**Background:**

In Sub Saharan Africa, there has been limited research on instruments to identify specific mental disorders in children in conflict-affected settings. This study evaluates the psychometric properties of three self-report scales for child mental disorder in order to inform an emerging child mental health programme in post-conflict Burundi.

**Methods:**

Trained lay interviewers administered local language versions of three self-report scales, the Depression Self-Rating Scale (DSRS), the Child PSTD Symptom Scale (CPSS) and the Screen for Child Anxiety Related Emotional Disorders (SCARED-41), to a sample of 65 primary school children in Burundi. The test scores were compared with an external ‘gold standard’ criterion: the outcomes of a comprehensive semistructured clinical psychiatric interview for children according the DSM-IV criteria (the Schedule for Affective Disorders and Schizophrenia for School-Age Children – K-SADS-PL).

**Results:**

The DSRS has an area under the curve (AUC) of 0.85 with a confidence interval (c.i.) of 0.73–0.97. With a cut-off point of 19, the sensitivity was 0.64, and the specificity was 0.88. For the CPSS, with a cut-off point of 26, the AUC was 0.78 (c.i.: 0.62–0.95) with a sensitivity of 0.71 and a specificity of 0.83. The AUC for the SCARED-41, with a cut-off point of 44, was 0.69 (c.i.: 0.54–0.84) with a sensitivity of 0.55 and a specificity of 0.90.

**Conclusions:**

The DSRS and CPSS showed good utility in detecting depressive disorder and posttraumatic stress disorder in Burundian children, but cut-off points had to be put considerably higher than in western norm populations. The psychometric properties of the SCARED-41 to identify anxiety disorders were less strong. The DSRS and CPSS have acceptable properties, and they could be used in clinical practice as part of a two-stage screening procedure in public mental health programmes in Burundi and in similar cultural and linguistic settings in the African Great Lakes region.

## Background

Global mental health researchers often use brief self-rating questionnaires to screen for DSM-IV disorders [1]. These instruments require minimal time and limited or no clinical expertise and training. They are, therefore, often recommended to be used in school, community and research settings to screen for symptoms of mental disorders [2]. In low-resource settings with extremely limited numbers of mental health professionals, a simple means to identify people with probable mental health problems may constitute an important component to develop a public mental health programme.

We have reported earlier on the development of the Child Psychosocial Distress Screener (CPDS), a screening instrument to identify children with high levels of psychosocial distress ^a^[3,4] in order to guide the triage of psychosocially affected children in situations of massive organized violence [5,6]. Children who scored above threshold on the CPDS were offered group-based psychosocial interventions [7]. However, the instrument does not differentiate between ‘nondisordered distress’ and ‘disordered distress’. In order to identify the children in need of more specialized interventions, instruments are required to screen for specific disorders such as depression, anxiety disorders or posttraumatic stress disorder (PTSD).

Screening questionnaires to detect child mental disorders are usually developed and validated in populations in high-income countries, and norm scores are commonly derived from research with western populations [8]. In a new context, instruments may have different psychometric properties [9,10]. Contexts vary in the extent to which symptoms are experienced and expressed. Uncritical use of self-report scales may lead to figures that skew prevalence rates of disorder and conflate mental disorder with subthreshold disorders or normal (‘nondisordered’) stress reactions in the face of loss and adversity [11,12]. Moreover they may measure grief reactions rather than specific mental disorder [13].

Ideally, validation of disorder-specific self-report questionnaires is recommended in new contexts [14]. This is certainly important in complex humanitarian emergencies with populations suffering from high levels of contextual distress in which uncritical use of self-report questionnaires may lead to inflated estimations of mental health disorders [11,15,16]. However, clinical validation in postconflict settings is not commonly done [17,18]. In Sub Saharan Africa, several validation studies with self-report questionnaires on mental problems have been carried out, mostly in adult populations [19-25]. Only a few African validation studies have been carried out with adolescents and youths [26-28] and children [29-31].

Many questionnaires focus on a particular category of mental disorders, for example depressive disorders or PTSD. Such questionnaires can be used to screen for mental disorders that are seen as priority in a given context. There is however debate in the literature about the usefulness of distinguishing between the various depressive and anxiety disorders on a population level or the level of primary health care. Some argue that, in unspecialized settings, designating cases as a ‘pure’ depressive episode or a ‘pure’ anxiety disorder may not be necessary because symptoms of both anxiety and depression are present in many cases, even if they are insufficient to support a full diagnosis in one of the categories [32,33]. Goldberg introduced the term ‘common mental disorder’ to denote any depressive or anxiety disorder (including PTSD) [34,35]. In Africa, the term ‘common mental disorder’ has been used, for example in adult populations in Zimbabwe [36,37] and Ethiopia [38]. For children in Africa, the concept of ‘common mental disorder’ has not been not been widely used, and there has been no research into a single questionnaire to identify children with mental disorder in need of assistance.

Our study took place in Burundi, a country that, since its independence in 1962, has experienced recurrent cycles of severe interethnic violence [39]. In 1993, after the assassination of the first democratically elected president, a civil war broke out that caused the death of an estimated 300,000 people and the displacement of many more [40]. Since peace agreements were signed in 2003, violence has significantly decreased, except in the three northwestern provinces (Bujumbura Rural, Bujumbura Mairie and Bubanza) where rebel groups remained active. In these three provinces, the nongovernmental organization (NGO) HealthNet TPO implements a community- and school-based psychosocial and mental health programme for children.

In the current paper, we explore the psychometric properties of three well-known self-report questionnaires for child psychiatric disorders in a sample of Burundian schoolchildren: the Depression Self-Rating Scale (DSRS) for depressive disorders [41], the Child PTSD Symptom Scale (CPSS) for PTSD [42] and the Screen for Child Anxiety-Related Emotional Disorders (SCARED) for anxiety disorders [43]. These questionnaires were translated in Kirundi, the local language of Burundi. The paper focuses on assessment of criterion validity by comparing the scores on the questionnaires with a ‘gold standard’, the presence of mental disorder as established through a semistructured clinical interview for child psychiatric disorder according to DSM-IV criteria. We also explored how well the three self-report questionnaires would be able to identify ‘any common mental disorder’ in children.

## Methods

### Design and participants

The sample consisted of children from three schools in the three provinces where HealthNet TPO implemented its school-based mental health programme. The schools were randomly chosen, using a random number generator, from a list with all schools provided by the Ministry of Education. In each school, the research assistant made a list of all children in the 4th or 5th grade and a random sample was drawn, again using a random number generator. In this way, 49 children aged from 10 to 15 years were randomly selected and invited to participate. We deliberately chose students from higher classes in school because these were the children targeted in the intervention programme. In order to gain a larger sample of probable cases of mental disorder, the sample was complemented with a random sample of 16 children from these same schools who had been identified in the psychosocial care programme and who received individual psychosocial care. Data were collected between January and May 2006. The demographic characteristics of the sample are described in Table 1.

**Table 1:** Descriptive statistics of the sample (n = 65)

Sex	Male	% or number
	Female	45%
Class	4th class	83%
	5th class	17%
Mean age		12.8 (SD 1.3)
Father has died		26%
Mother has died		14%
Number of siblings that are alive		4.2 (SD 1.9)
Number of siblings that have died		1.8 (SD 2.2)
Children with at least one deceased sibling		60%
Having witnessed relatives been killed		32%
Having witnessed unknown people been killed		46%
Family situation	Lives with both parents	51%
	Lives with one parent	40%
	Does not live with parents	9%
Has been displaced		92%
House has been burnt		69%

The assessment for each child was done in two steps that were both carried out on the same day. The first step consisted of administering three self-report questionnaires. A Burundian research assistant read each question and asked the child to respond following the available response format (30–45 minutes). These research assistants had a Bachelors degree in social science and had been trained in quantitative data collection during a five-week period.

The second step was a psychiatric assessment by an expatriate research psychiatrist (PV) and a Burundian psychologist, who were both blinded to the results of the three self-report questionnaires; further, for the 16 children who received individual counselling, they did not know the type of problems that the child had, nor did they study the case files. This psychiatric assessment took place in an empty classroom, after school time with no other persons present apart from the child and the two clinician-researchers. At the time of the study, the research psychiatrist had lived and worked in Burundi for around two years, spoke fluent French, had some basic understanding of local Kirundi concepts and was involved in clinical work, supervision and training of Burundian mental health professionals. The second member of the assessment team was one of two Burundian psychologists who took turns. They had worked with the psychosocial project for children of HealthNet TPO since 2005 and were trained in psychosocial assessments with children. The Burundian psychologist took the lead in asking questions and acted as a translator for the research psychiatrist. The psychiatric assessment team (psychiatrist and psychologist) had been trained for seven days in the use of the Schedule for Affective Disorders and Schizophrenia for School-Age Children (K-SADS-PL) by an Italian child psychiatrist (PF) and his Burundian research assistants who had extensive experience with the instrument.

The clinical assessment started with a general section that took between 15 and 45 minutes, in which the two interviewers built rapport with the child and obtained information about biographical events (including death or sicknesses of siblings or parents), the migration history and the current life situation of the child. In the Burundian context, children are not used to speak about their emotional life and are often quite reticent at first contact. Therefore, an introduction phase was of importance to make the child feel comfortable in front of two unknown adults.

Subsequently, a semistructured interview was held with the help of the K-SADS-PL that had been translated into Kirundi. The psychiatric assessment took on average 105 minutes (between 45 and 200 minutes). When the responses of the child were not sufficiently clear after the interview with the child, additional information about the functioning of the child was obtained from the teacher. Both the psychiatrist and psychologist independently rated the scores on the K-SADS-PL and made additional notes during the interview. The scores and notes were reviewed after the interview; in case of different ratings on an item, the assessors discussed this to come to a consensus agreement; here, they took into account the cultural relevance of presented symptoms and their severity, using cultural information regarding the Burundian setting and contextual knowledge about the child’s family background and daily life. For example, the K-SADS probing questions for separation anxiety disorder contain questions about ‘fears of harm befalling an attachment figure’ such as ‘*Has there ever been a time when you worried about something bad happening to your parents? Like what? Were you afraid of them being in an accident or getting killed? Were you afraid that they would leave you and not come back? How much did you worry about this?*’ In evaluating whether the child’s responses were pathological, it proved essential to include a general knowledge of the violence in the area and the particular life history of the child. Other examples include the evaluation of sleeping with the mother in the same bed, or children who reported to be afraid in the dark. In such instances information about prevailing cultural norms in Burundi are important and the discussions between the Burundian and expatriate mental health experts proved to be very helpful.

The study was part of a multisite study on how secondary-school-based interventions affect the psychosocial wellbeing of violence-affected children in Burundi, Indonesia, Nepal, South Sudan and Sri Lanka [44,45]. The research proposal, including the elements reported in this study, was reviewed and approved by the Medical Ethics Committee of the VU University Medical Centre in Amsterdam, the Netherlands. A written approval was obtained on 28 March 2006.

Before starting research activities, meetings were organized in schools, with parents, teachers and principals, to explain the research purposes, including the selection method, and to obtain informed consent. The objectives of the study were read out to each participant, individual informed written consent from children and their caregivers was obtained before starting the interview, and confidentiality was assured by explaining to participants about procedures of data storage and anonymity. Data collection procedures were consistent with the Declaration of Helsinki [46].

### Instruments

We translated all instruments from English into Kirundi, using a five-step procedure for cross-cultural translation [47]: (1) translation from English into Kirundi, and lexical back-translation; (2) review by a bilingual mental health professional; (3) evaluation of items in focus group discussions of Burundian children from the study area; (4) blind back-translation from Kirundi into English by a bilingual Burundian psychologist who was unfamiliar with the original version, and comparison of the back-translation with the original; (5) pilot testing in a Burundian school.

#### DSRS

The DSRS is an 18-item questionnaire designed to identify symptoms of depression among children and adolescents [41]. The items refer to the frequency of self-reported symptoms in the past week utilizing a three-point scale response format including ‘never’, ‘sometimes’ and ‘most of the time’. The major advantages of the DSRS are its very simple language, brevity and ease of use and scoring [48]. Internal consistency was good in samples of British children (α = 0.86) [41] and Swedish children (α = 0.88) [49]. However, in the United Kingdom, the DSRS showed only moderate discrimination between depressed and nondepressed children in the diagnosis of depression in 93 children (aged 8–16 years) attending a university child psychiatry department, with around of 25% of the children misclassified [50].

The scale has been used among children affected by natural disaster or armed conflict in several low- and middle-income countries (LMICs) [45,51-66] and among refugee children who migrated to high-income countries [67,68]. To our knowledge, it has never been validated for children in Africa. It was designed to be a written test, but in nonwestern populations it is often used orally [67]. In western populations, a cut-off score between 13 [69] and 15 [70] was optimal to discriminate depressed from nondepressed children. In Indonesia, the optimal cut-off point (19.5) was higher than in western populations, with an area under the curve (AUC) of 0.76 [55]. In Nepal, the optimal cut-off point was 14 and the AUC was 0.82 [71].

#### CPSS

The CPSS is a 17-item questionnaire to detect symptoms of PTSD in children [42] is the child version of the Posttraumatic Diagnostic Scale, a widely used and well-validated instrument for assessment of PTSD severity and diagnosis in adults [72]. It measures symptoms in the three clusters of DSM–IV category of PTSD (re-experiencing, avoidance and arousal) and thus provides a PTSD symptom severity score. Symptom items are rated on a four-point frequency scale (0 = ‘not at all’ to 3 = ‘five or more times a week’).

The scale has been used in various LMICs among children and adolescents exposed to collective violence [45,54,73,74]. In samples of trauma-exposed children in the US and war-affected children in Nepal, the internal consistency was high (α between 0.81 and 0.89) [54,72,75,76]. The standard cut-off point is 11, with American children having a sensitivity of 95% and a specificity of 96% [72]. In a conflict-ridden area of Indonesia, the psychometric properties were different, with a considerable higher optimal cut-off point of 17 (AUC = 0.71), while in Nepal the optimum cut-off score was 20 (AUC = 0.77) [71].

#### SCARED-41

The SCARED is a self-report instrument designed as a screening tool for anxiety disorders in children and adolescents according to the DSM IV-TR classification [43]. The severity of symptoms is rated using a three-point response format, ranging from 0 (‘not true’) to 2 (‘often true’). The questionnaire has five subscales: panic disorder; generalized anxiety disorder; separation anxiety disorder; social anxiety disorder; and school anxiety. Due to difficulties of the scale in discriminating between social anxiety and other anxiety disorders, its developers have adapted its original 38-item version into a 41-item version, the SCARED-41, which was used in our study [77]. Factor analysis with the SCARED-41 in clinical samples of American and Dutch children showed a five-factor solution confirming the five subscales, with each factor showing good internal consistency (α ranging between 0.78 and 0.87) [77,78]. The five-factor structure was more or less also found in nonclinical populations of South African children [79]; however, in a multiethnic primary-care population, the factor structure was less robust across ethnic and gender subgroups [80]. A recent meta-analysis of 25 studies on the SCARED, with only three done in LMICs (two in South Africa [79,81] and one in China [82]), showed good psychometric properties and suggested that the scale could be used in various cultural settings to screen for DSM-IV anxiety disorders [83]. Since then, the psychometric properties of the SCARED-41 have been studied in Brazil [84] and Iran [85], confirming the five-factor structure and demonstrating good convergent validity and discriminate validity. Apart from the validation studies by the developers of the SCARED [43,77,86], only two studies assessed concurrent validity using a structured clinical psychiatric interview as gold standard. In Brazil, the total score of the SCARED-41 in a community sample of students aged 9–18 had an optimum cut-off point of 22 (AUC 0.73, sensitivity 52%, specificity 82%) [87]. In a sample of children and adolescents attending a psychiatric clinic in Lebanon, the cut-off score that maximized both sensitivity and specificity was 26 (AUC 0.63, sensitivity 66% and a specificity of 56%) [88].

#### K-SADS-PL

Psychiatric diagnosis was established using the K-SADS-PL, a comprehensive semistructured clinical interview designed to identify Axis I mental disorders in children according to DSM-IV criteria [89]. It uses probing questions to establish the presence or absence of a DSM-IV symptom (present/subthreshold/absent). Its application procedure mirrors closely the clinical diagnostic process that is employed by trained clinicians. Symptoms are rated in a format that allows for rephrasing and asking additional clarifying questions, an aspect that allows flexibility when the instrument is used in a cultural context that differs from its source. The instrument has been used widely in clinical research and practice in western settings but, to the best of our knowledge, this is the first translation to an African context. The translation was done by a group of Italian, Dutch and Burundian researchers and followed the same translation procedure as described for the DSRS, CPSS and SCARED-41 [90]. The Kirundi version of the K-SADS-PL assists clinicians in the diagnosis of depressive disorders, anxiety disorders including PTSD, alcohol use and addiction, ADHD, enuresis nocturna and oppositional defiant disorder. It does not include the section on psychotic disorders, but in each interview, basic questions on delusions and hallucinations were asked, together with careful observation of the child during the whole interview, to exclude the presence of psychosis. The section on eating disorders in the K-SADS-PL was not included because of the limited relevance in the Burundian setting (with high rates of malnutrition). The screening questionnaire of this version of the K-SADS-PL has a total of 49 items to be scored. When one or more items in the screening questionnaire scores positively, a corresponding supplement is to be administered.

Interrater reliability between the multicultural assessment team of this research (a Dutch psychiatrist [PV] with a Burundian psychologist) and the team that trained them (an Italian child psychiatrist [PF] with extensive experience with the K-SADS-PL in Italy and Burundi, and his Burundian research assistant) in a convenience sample of 11 patients in Burundi was good: in 10 cases, the two raters came to exactly the same DSM-IV classification, while in one case the diagnosis differed slightly: a depression not otherwise specified versus a mild depression. On item level, kappa values were substantial to good (0.75–1.00).

#### Statistical analysis

Internal consistency of the three questionnaires was measured by Cronbach’s alpha. The scores on the screening questionnaires in relation to ‘psychiatric caseness’ (the clinical psychiatric diagnosis with the K-SADS-PL) were evaluated using Receiver Operating Characteristic (ROC) analysis. ROC analysis plots the diagnostic sensitivity against ‘1 minus specificity’ of each value of a dimensional screening scale. For each ROC curve, the AUC was calculated, indicating the accuracy in detection of caseness. Positive predictive value (PPV) and negative predictive value (NPV) for the screening questionnaires were calculated at various cut-off points. We analyzed the discriminative diagnostic capacity of the scales by means of ROC analysis of the different scores on the three questionnaires against the appropriate clinical diagnosis as derived from the psychiatric interview through the K-SADS-PL and against ‘any common mental disorder’. As a means to measure overall diagnostic effectiveness of a test using a specific cut-off point, we also calculated Youden’s index (J): the sum of sensitivity + specificity minus 1 (where sensitivity and specificity are calculated as proportions). This index ranges between 0 and 1, with values close to 1 indicating that the effectiveness of the test is relatively large [91].

## Results

Many of the children in the sample had experienced violence and death (see Table 1): 92% had been displaced, 69% had their house burnt. 60% had at least one sibling who had died. 17 children (26%) had lost their father, in 13 cases often due to war violence, and nine children (14%) had lost their mother (five due to war violence).

The psychiatric assessments with K-SADS-PL in our sample (n = 65) identified 28 children (43%) with a common mental disorder such as depressive disorder, PTSD and/or another anxiety disorder. See Table 2 for a breakdown of diagnoses.

**Table 2:** Psychiatric diagnosis according to K-SADS-PL

**Diagnosis**	**n = 65**	**%**
**Depressive disorders**	**12**	**18**
Depression	10	15
Adjustment Disorder with depressive symptoms	2	3
**Posttraumatic stress syndromes**	**15**	**23**
PTSD	7	11
PTSD subsyndromal cases	8	12
**Other anxiety disorders**^**1**^	**15**	**23**
Separation Anxiety Disorder	5	8
Panic Disorder	2	3
Obsessive Compulsive Disorder	1	2
Anxiety NOS	12	18
**Any common mental disorder**^**2**^	**28**	**43**
**No diagnosis**	37	57

K-SADS-PL, Schedule for Affective Disorders and Schizophrenia for School-Age Children; NOS, not otherwise specified; PTSD, posttraumatic stress disorder.

^1^Numbers of the specific anxiety disorders do not add up because some children had more than one anxiety disorder.

^2^Numbers of ‘depressive disorders’, ‘posttraumatic stress syndromes’ and ‘other anxiety disorders’ do not add up because some children had more than one disorder.

### Internal consistency

In our sample the total scores of three scales had a high internal consistency (DSRS: α = 0.85; CPSS: α = 0.90; SCARED-41: α = 0.92). For the subscale scores of the CPSS, internal consistency was good (re-experiencing subscale α = 0.84; avoidance/numbing subscale, α = 0.79; and hyperarousal subscale, α = 0.77). Internal consistency was high in four of the five subscale scores of the SCARED-41 (panic/somatic subscale, α = 0.86; social phobia subscale, α = 0.76; generalized anxiety subscale, α = 0.71; separation anxiety disorder subscale, α = 0.70). Only the scores for the SCARED-41 subscale for school phobia did not show sufficient internal consistency (α = 0.49).

### Receiver operating characteristic curve analysis: sensitivity and specificity

**Figure 1:**
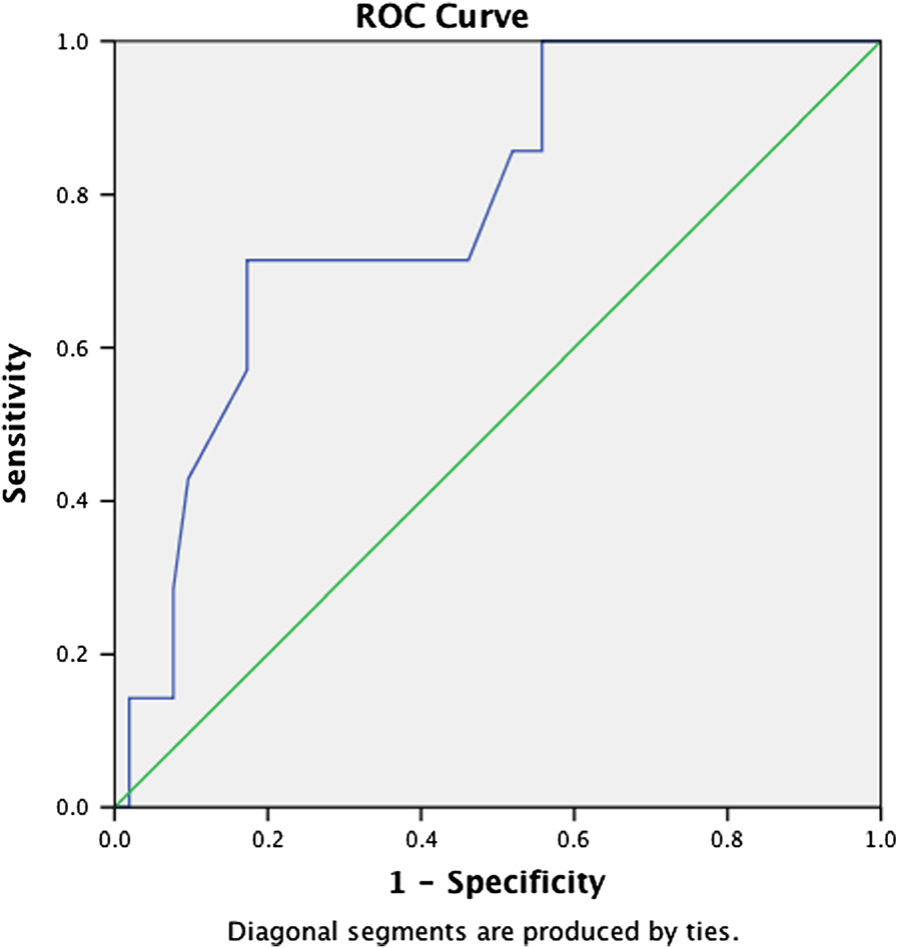
ROC curve of the CPSS (PTSD).

**Figure 2:**
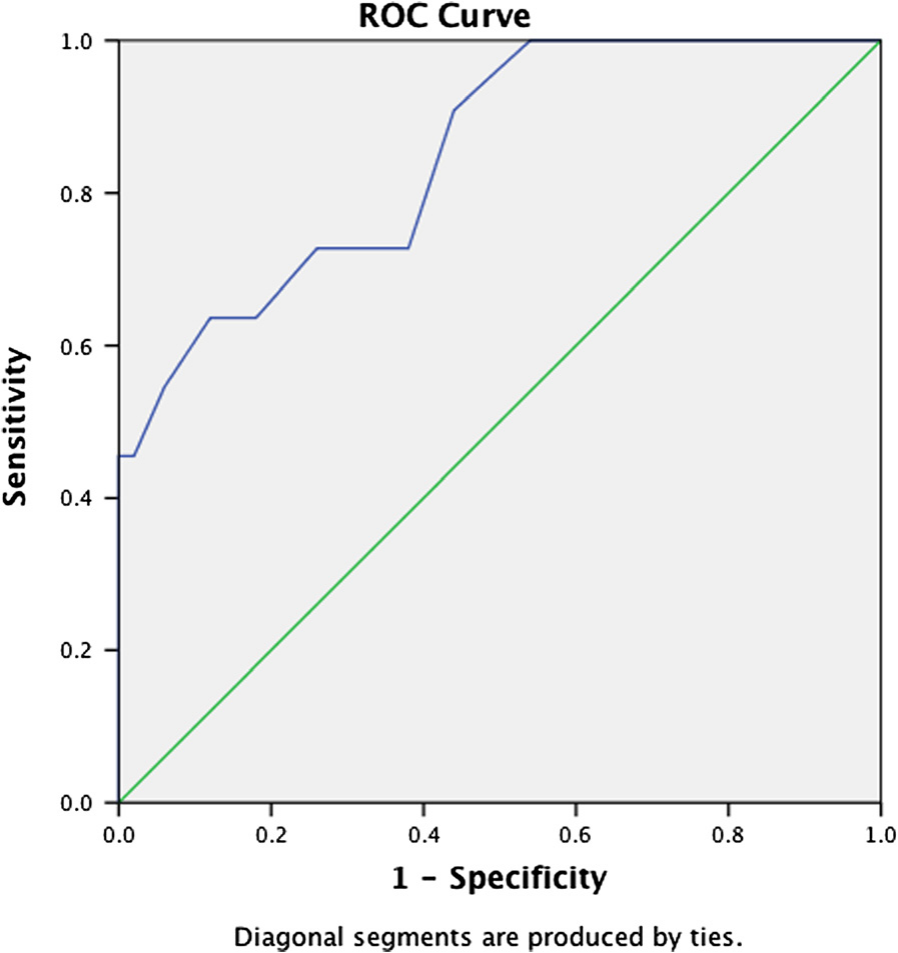
ROC curve of the DRSR (depressive disorders).

**Figure 3:**
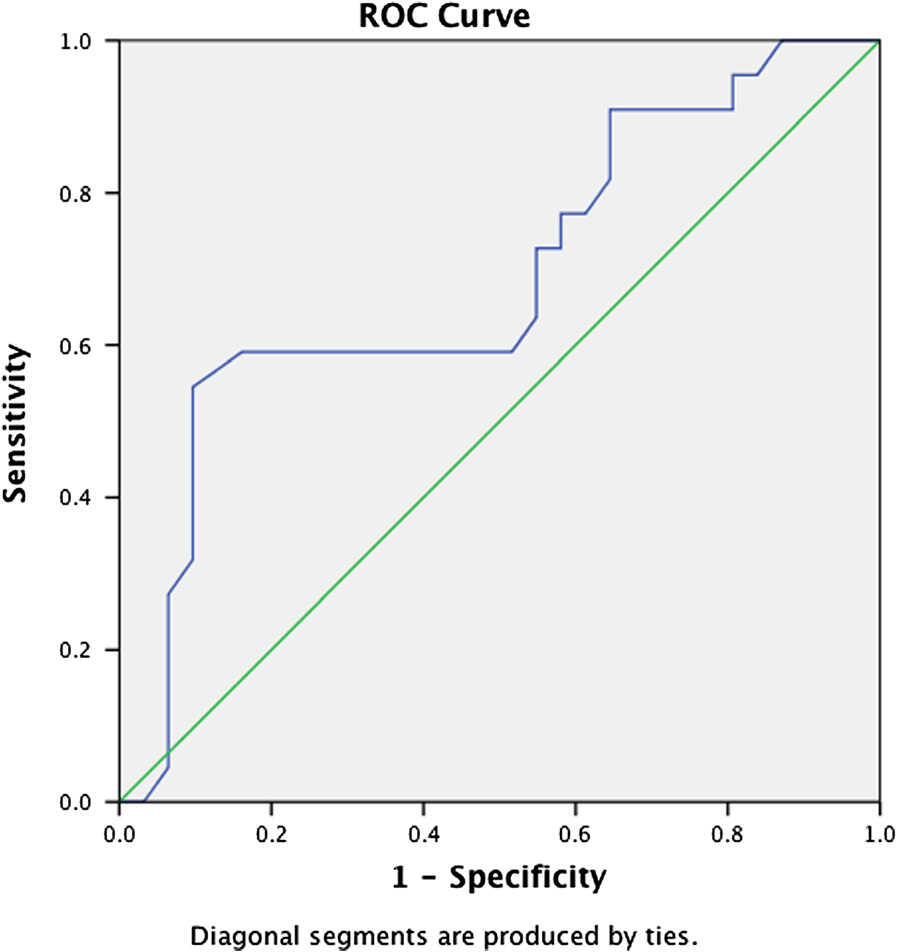
ROC curve of the SCARED-41 (anxiety disorders including PTSD).

For the scores on each of the three self-rating scales, we created an ROC curve by plotting the fraction of true positives out of the positives against the fraction of false positives out of the negatives, at various cut-off points (see Figures 1, 2, 3). Performances of the CPSS (measuring posttraumatic stress [AUC 0.78]) and of the DSRS (measuring depressive disorders [AUC 0.85]) were good, while the SCARED-41, measuring anxiety disorders including PTSD, performed less well (AUC 0.69).

### Performance of the instruments at various cut-off points

Tables 3, 4, 5 present the performance at various cut-off points of the CPSS, DSRS and SCARED-41 to detect PTSD, depression and anxiety disorders, respectively. For each cut-off point, we calculated the overall diagnostic effectiveness (J), i.e. the sum of specificity and specificity minus 1. For the CPSS, measuring posttraumatic stress, the highest J was reached at a cut-off score of 26. For this cut-off point, sensitivity was 71% and specificity 83%. The PPV was 0.36 and the NPV was 0.96. If, instead, the standard cut-off point of 11 that has been found in western research had been used, the test parameters in this population would have worsened drastically: PPV 0.15 and NPV 1.00. For the DSRS, measuring depressive disorders, the cut-off point with the highest J was 19 (sensitivity 64%, specificity 88%, PPV 0.54, NPV 0.92). For the SCARED-41, measuring anxiety disorders including PTSD, the highest J was reached at a cut-off point of 44 (sensitivity 55%, specificity 90%, PPV 0.80, NPV 0.74).

**Table 3:** Properties of the Kirundi version of the CPSS to detect children with PTSD

**AUC (95% c.i.)**	**Cut-off score**	**Sensitivity (%)**	**Specificity (%)**	**PPV**	**NPV**	**J**
	11	100	22	0.15	1	0.21
	14	100	35	0.17	1	0.35
0.78 (0.62–0.95)*	17	86	44	0.17	0.96	0.30
	21	71	60	0.19	0.94	0.30
	24	71	79	0.31	0.95	0.52
	26	71	83	0.36	0.96	0.54
	28	57	83	0.31	0.93	0.40

AUC, area under curve; CPSS, Child PTSD Symptom Scale; J, Youden’s index; NPV, negative predictive value; PPV, positive predictive value, PTSD, posttraumatic stress disorder.

*= p < 0.05.

**Table 4:** Properties of the Kirundi version of the DSRS to detect children with depression

**AUC (95% c.i.)**	**Cut-off score**	**Sensitivity (%)**	**Specificity (%)**	**PPV**	**NPV**	**J**
0.85 (0.73–0.97)**	13	91	56	0.31	0.97	0.47
	15	73	66	0.32	0.92	0.39
	17	64	82	0.44	0.91	0.46
	19	64	88	0.54	0.92	0.52
	21	46	98	0.84	0.89	0.44

AUC, area under curve; DSRS, Depression Self-Rating Scale; J, Youden’s index; NPV, negative predictive value; PPV, positive predictive value.

**p < 0.01.

**Table 5:** Properties of the Kirundi version of the SCARED-41 to detect children with anxiety disorders including PTSD

**AUC (95% c.i.))**	**Cut-off score**	**Sensitivity (%)**	**Specificity (%)**	**PPV**	**NPV**	**J**
0.69 (0.54–0.84)*	22	91	19	0.44	0.75	0.10
	25	91	29	0.48	0.82	0.20
	29	82	36	0.47	0.73	0.17
	33	73	45	0.48	0.70	0.18
	36	59	52	0.46	0.64	0.11
	41	59	74	0.62	0.72	0.33
	44	55	90	0.80	0.74	0.45
	49	46	90	0.77	0.70	0.36

AUC, area under curve; J, Youden’s index; NPV, negative predictive value; PPV, positive predicted value; PTSD, posttraumatic stress disorder; SCARED-41, Screen for Anxiety-Related Emotional Disorder, 41-item version.

* = p < 0.05.

We also used the capability of the three questionnaires to detect any ‘common mental disorder’ (which we define as any depressive disorder and/or any anxiety disorder, including PSTD). Here, all questionnaires performed moderately (CPSS: AUC = 0.71; DSRS: AUC = 0.73; SCARED-41: AUC = 0.75) (see Table 6).

**Table 6:** Properties of self-report questionnaires to detect ‘common mental disorders’

**Instrument**	**AUC (95% c.i.))**	**Cut-off score**	**Sensitivity (%)**	**Specificity (%)**	**PPV**	**NPV**	**J**
CPSS	0.71 (0.58–0.84)**	11	89	26	0.52	0.73	0.15
		14	82	42	0.56	0.72	0.24
		16	79	55	0.61	0.74	0.33
		19	64	64	0.62	0.67	0.29
		21	57	68	0.61	0.64	0.25
		26	39	90	0.79	0.62	0.30
DSRS	0.73 (0.60–0.85)**	12	75	49	0.55	0.70	0.24
		15	57	73	0.64	0.67	0.30
		18	46	94	0.87	0.67	0.40
		21	18	97	0.84	0.58	0.15
SCARED-41	0.75 (0.61–0.88)**	36	62	56	0.57	0.60	0.17
		41	62	82	0.76	0.69	0.43
		43	55	96	0.93	0.68	0.51
		45	50	96	0.93	0.67	0.46

AUC, area under curve; CPSS, Child PTSD Symptom Scale; DSRS, Depression Self-Rating Scale; J, Youden’s index; NPV, negative predictive value; PPV, positive predicted value; PTSD, posttraumatic stress disorder; SCARED-41, Screen for Anxiety-Related Emotional Disorder, 41-item version.

**p < 0.01.

## Discussion

Concurrent validation of the test scores on the DSRS and the CPSS against a clinical gold standard showed reasonably good psychometric properties, while the properties of the SCARED-41 were not satisfactory. The relatively weak performance of the SCARED-41 may be related to the fact that this instrument, in contrast to the DSRS and the CPSS, targets not a single diagnostic category but various categories of anxiety disorder. Screening instruments that mirror closely the diagnostic criteria of a specific disorder against which they are validated will perform better in singling out that disorder, while instruments that are meant to identify a broad range of disorders will be less able to discriminate between disorders. Moreover, the combination of anxiety disorders covered by the SCARED-41 has a poor fit with local concepts of and idioms for anxiety in Burundi, and therefore that tool does not capture the construction and elaboration of anxiety in the local cultural setting [92].

The properties of the Kirundi versions of the DSRS and CPSS to detect depression and PTSD are good. However, to improve utility, the cut-off points of all self-report questionnaires had to be raised (see Tables 3 and 4) to reach acceptable psychometric properties. If we had used the standard cut-off score of 15 for the DSRS as established for British children, the utility of the scale would have deteriorated considerably (PPV 0.54 and NPV 0.92 for optimum cut-off point in this research and PPV 0.32 and NPV 0.93 for the standard cut-off point) [41]. Similarly, for the CPSS and for the SCARED-41, using the standard cut-off points as established in American children would have given significant overestimations of children with mental disorder.

In other settings affected by collective violence, higher cut-off points have been found for self-report questionnaires [28,93]. We postulate that this may be related to expressing overall high nonspecific distress in the research areas where the level of everyday violence was high, and many of the interviewed children lived in adverse circumstances. Another reason for high cut-off points may be related to the lack of validity of universal classificatory constructs in the local setting caused by symptoms that overlap with idioms of distress, as has been found elsewhere in Africa [94,95].

When used to identify any common mental disorder in children, all three questionnaires performed reasonably, with the SCARED-41 demonstrating the best properties.

### Making screeners useful within low resource settings

However, acceptable psychometric properties are by themselves not enough to justify a broad use in general health practice. While our results are promising, we are cautious about overt optimism that introducing mental health screening tools for use by nonspecialists or lay workers by themselves would make ‘a dramatic contribution to the health sector’s ability to identify those in need of mental health support’ [96]. The utility of scales in actual practice may be decreased by practical and logistical factors, such as the time needed to administer the scale and the ease of interpretation of results, as well as by ethical concerns, related to the identification of children with mental disorders without the ability to provide them with adequate treatment [97].

Therefore, we argue that screening questionnaires can best be used within a multileveled system of care. In Burundi, the project by HealthNet TPO uses a community mental health approach that has both curative and preventive components. In our child psychosocial programme, we used a generic psychological distress-screener as the first step to identify children who are in need of psychosocial support without differentiating between those with and without mental disorder. The instrument in this *universal* screening, the CPDS, can be administered by trained community workers or classroom assistants, requiring less than 5 minutes per child. This is followed by a second step of *selective* screening in which instruments to identify children with a probable mental *disorder,* such as the CPSS and DSRS can be administered by trained lay persons, taking around 20–30 minutes per child. Screening would routinely have to be followed by clinical assessment of all screen positives to reduce the proportion of false positives [98]. This will require targeted training of general health care workers about mental disorders in order to help them to identify people with mental disorders *during* the clinical encounter [99]. The World Health Organization promotes this training approach, a component of task sharing, through its Mental Health Gap Action Programme, which includes an Intervention Guide for Mental, Neurological and Substance Use Disorders in Non-Specialized Health Settings [100]. Within public health programmes to build the capacity of primary-care providers to manage mental disorders in children and adolescents [101], screening instruments may also be useful to enhance the ability of care providers to identify mental disorders in children and adolescents once those instruments are validated and calibrated for the settings in which they are to be used.

### Strengths and limitations

The study has several strengths. The major strength of our method is the use of a structured clinician-administered child psychiatric interview. Our study is one of the few validation studies in Africa using a full clinical child psychiatric interview. Another strength of the study is that it was conducted in the context of an ongoing service delivery programme. All children were recruited from schools where HealthNet TPO implemented a school-based mental health programme, and 16 of them had already received individual psychosocial care. Some of the other children had participated potentially in resilience groups or other activities. Hence, all children were familiar with the activities of the NGO and had presumably gained trust in the persons associated with HealthNet TPO. This might be a contributing factor for the readiness of the children to disclose the presence of symptoms and other personal problems. Usually the Burundian culture highly values keeping personal problems private, avoiding potential conflicts or admission of weakness or illness.

This study has several limitations. First, the gold standard that we used, a clinical psychiatric assessment using a semistructured interview, was not performed by a local Burundian psychiatrist. During the time of the research, there was no Burundian psychiatrist active in the country. In order to account for contextual and culture-specific factors that may influence psychiatric diagnosis, the psychiatric assessment was carried out by a bicultural team consisting of a Burundian psychologist and an expatriate psychiatrist.

Second, in this paper we focused on a form of criterion validity: we assessed the concurrent validity by comparing scores on three self-report questionnaires with a standardized clinical interview as the external criterion or gold standard. We did not explore other aspects of validity, such as convergent and discriminant validity. We also explored only one aspect of reliability (internal consistency), while we did not assess test–retest reliability. This study reports on the first step in a staged process of validation. For future use of the CPSS, DSRS and SCARED-41 tools, further studies on the convergent or discriminative validity are recommended.

Third, in hindsight, we regret that we have not used emic local categories of mental distress in our evaluation [92,102]. While many words and phrases could be successfully translated into Kirundi, there were special challenges to differentiate between expressions of feelings that are close to each other. As is noted for other Bantu languages [103], Kirundi has relatively few words to differentiate directly between dysphoric emotional states and this may particularly have affected the diagnostic accuracy of the SCARED-41, which contains various terms related to anxiety. Within a semistructured diagnostic interview, this problem is less obvious because the interviewer can use metaphors and descriptions to ensure the person understands the term [104].

Fourth, this study was done in the context of ongoing service delivery in Burundi, which is both a weakness and a strength: while this is, in general, a desirable approach in order to establish efficient and sustainable assistance, the fact that the children knew the activities of the NGO for a long time may have made them feel more comfortable to discuss their personal experiences. This may have influenced the results, and this could theoretically compromise the generalizability of the results regarding the cut-off scores.

Finally, validating with a gold standard is time consuming and, considering the limited resources, we could therefore interview only a relatively small number of children for the clinical validation. Involving more cases would have improved the statistical power of the study. Only seven children had the diagnosis of PTSD, for example. Moreover, when three instruments are administered to 65 people, the chance that at least one instrument will register poorer-than-usual psychometric properties is relatively high. Therefore, future research with self-report questionnaires for children in Africa should be done with larger samples to corroborate our outcomes and explore whether the cut-off points need to be adapted.

## Conclusion

Brief self-report questionnaires are often used in research to estimate the prevalence of psychiatric disorders in African children. Our findings underline the need for a clinical validation of brief self-report questionnaires before meaningful interpretation of scores can be done. We concur with Ertl et al. [26] and de Jong and van Ommeren [105] who caution against the application of ad hoc translated clinical instruments without validation and adjustment of cut-off scores across different populations. For research on depression and PTSD, the DSRS and CPSS have acceptable psychometric properties in war-affected Burundian children, but the optimal cut-off points are considerably higher than in western norm populations. All three questionnaires, including the SCARED-41, have acceptable properties for use as a generic screener to identify ‘any common mental disorder’.

In our opinion, self-report questionnaires to identify mental disorders can best be given *clinical utility* by incorporating them within a specific multitiered system of care that requires a two-stage screening procedure.

## Endnotes

^a^The term ‘psychosocial’ is defined by the interaction between psychological factors and problems with the social context. With the word ‘psychosocial’, we refer not only to the subjective nature of the child’s experiences but also to the social nature of stressors, behavioural responses and contributions made to the community. It is thus a broad and rather a-specific concept [3,4].

## Abbreviations

AUC: Area under the curve; CPDS: Child psychosocial distress screener; CPSS: Child PTSD symptom scale; DSRS: Depression self-rating scale; K-SADS-PL: Schedule for affective disorders and schizophrenia for school-age children; LMIC: Low- and middle-income country; NGO: Nongovernmental organization; NPV: Negative predictive value; PPV: Positive predictive value; PTSD: Posttraumatic stress disorder; ROC: Receiver operating characteristic; SCARED-41: Screen for child anxiety-related emotional disorders, 41-item version.

## Competing interests

The authors declare that they have no competing interests.

## Authors’ contributions

PV participated in the design of study and in the data collection, executed the statistical analysis of the data and drafted the first version of the manuscript. IK participated in the design of study, supervised the statistical analysis and participated in the drafting of the manuscript. MJ participated in the design of study and in the drafting of the manuscript. PF participated in the data collection and in the drafting of the manuscript. JdJ participated in the design of study and participated in the drafting of the manuscript. All authors read and approved the final manuscript.

## Pre-publication history

The pre-publication history for this paper can be accessed here:

http://www.biomedcentral.com/1471-244X/14/36/prepub
